# Closing the Loop on Deep Brain Stimulation for Treatment-Resistant Depression

**DOI:** 10.3389/fnins.2018.00175

**Published:** 2018-03-21

**Authors:** Alik S. Widge, Donald A. Malone, Darin D. Dougherty

**Affiliations:** ^1^Department of Psychiatry, Massachusetts General Hospital and Harvard Medical School, Boston, MA, United States; ^2^Department of Psychiatry, Cleveland Clinic, Cleveland, OH, United States

**Keywords:** deep brain stimulation, neuromodulation, depression, electrophysiology, neuro-imaging, brain circuits, brain-computer interfaces

## Abstract

Major depressive episodes are the largest cause of psychiatric disability, and can often resist treatment with medication and psychotherapy. Advances in the understanding of the neural circuit basis of depression, combined with the success of deep brain stimulation (DBS) in movement disorders, spurred several groups to test DBS for treatment-resistant depression. Multiple brain sites have now been stimulated in open-label and blinded studies. Initial open-label results were dramatic, but follow-on controlled/blinded clinical trials produced inconsistent results, with both successes and failures to meet endpoints. Data from follow-on studies suggest that this is because DBS in these trials was not targeted to achieve physiologic responses. We review these results within a technology-lifecycle framework, in which these early trial “failures” are a natural consequence of over-enthusiasm for an immature technology. That framework predicts that from this “valley of disillusionment,” DBS may be nearing a “slope of enlightenment.” Specifically, by combining recent mechanistic insights and the maturing technology of brain-computer interfaces (BCI), the next generation of trials will be better able to target pathophysiology. Key to that will be the development of closed-loop systems that semi-autonomously alter stimulation strategies based on a patient's individual phenotype. Such next-generation DBS approaches hold great promise for improving psychiatric care.

## Introduction: circuit-directed treatments for circuit illnesses

The past 20 years have been promising and frustrating for psychiatry. We have new insight into circuits that are conserved across species and involved in mental illness, but that research has not yielded a substantial change in the treatment of mental disorders. New technologies for electrical and magnetic brain stimulation have renewed hope that we may be able to target and remediate those dysfunctional circuits (Lo and Widge, 2017). Implantable devices, although invasive, are particularly powerful, because they can limit their effect to a very small brain region or circuit. The leading example, deep brain stimulation (DBS) of the basal ganglia, has revolutionized the treatment of Parkinson disease and other movement disorders (Miocinovic et al., 2013). DBS delivers high-frequency electrical pulses that affect neural tissue in a roughly 0.5–1 cm diameter area around one or more active electrical contacts at the implant tip. Mood and anxiety symptoms are also linked to deep brain structures (Williams, 2016), raising the possibility that DBS may be more effective than non-invasive stimulation that affects only the cortical mantle. The history of successful stereotactic lesioning for severe depression and obsessive-compulsive disorder (OCD) patients further supports that theory (Park et al., 2006; Dougherty and Widge, 2017). DBS has thus been tested in multiple brain areas for severe depressive symptoms, with strong open-label responses and a mixed picture in randomized controlled trials.

**Figure 1:**
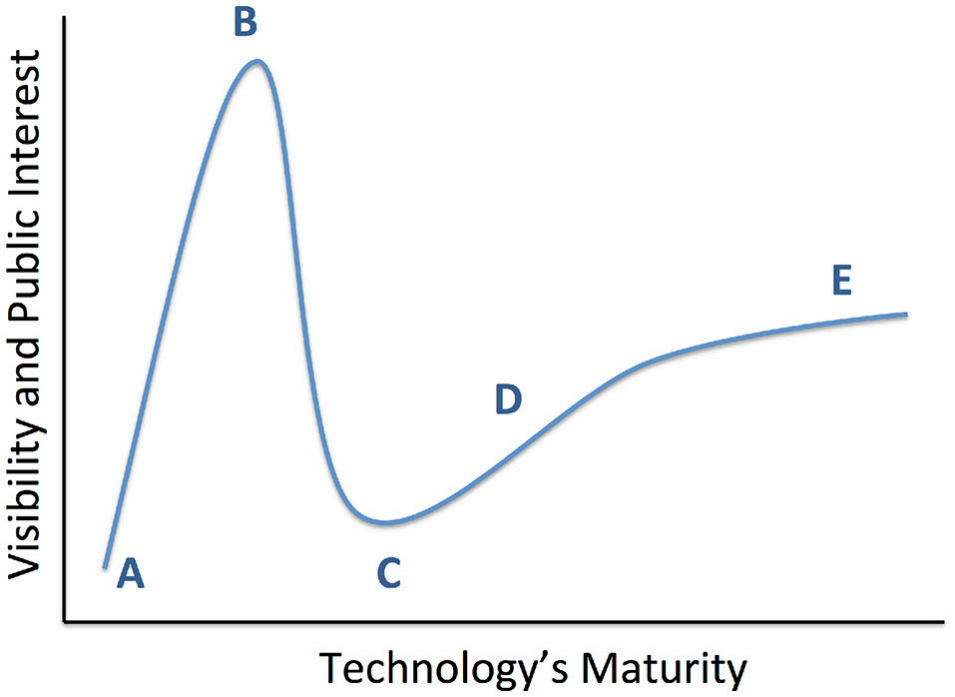
Conceptual diagram of technology maturation, originally proposed by the Gartner Group as the “hype cycle.” From a technology's inception **(A)**, early positive results propel it to a “peak of inflated expectations” **(B)**, where interest in the technology exceeds its maturity or its creator's understanding. This leads into a “valley of disillusionment” **(C)**, where early adopters become discouraged and may abandon the technology. A slower phase of progress climbs a “slope of enlightenment” **(D)**, where the lack of intense public attention enables more thoughtful development of robust solutions. This leads ultimately to a “plateau of productivity” **(E)** and readiness for wide adoption. We believe that recent studies have moved psychiatric DBS to the cusp between **(C,D)**.

We review the results of those studies, with an emphasis on three major randomized, double-blind, sham-controlled clinical trials (RCTs) conducted for treatment resistant depression (TRD) to date. This is a qualitative review; at present, these trials are the entirety of RCT evidence for DBS in TRD. The different brain structures involved and different trial designs make it premature to attempt any formal meta-analysis or search-based literature synthesis. The RCT results are inconsistent, with two not meeting their pre-specified efficacy targets. The clinical community has considered this evidence of DBS' poor efficacy. We argue that it represents a need for improvements in technology and clinical trial design. Observations during these clinical studies, paired with ancillary physiologic studies, demonstrate clear DBS effects beyond placebo in some patients with TRD. We suggest that psychiatric DBS, like many novel treatments, is progressing through a technology maturation cycle (Figure 1) or “hype cycle” (Gartner, Inc.^1^). In this model, early successes lead to a peak of unreasonable enthusiasm, followed by a “valley of disillusionment” as findings regress toward their true mean. The valley is followed by a “slope of enlightenment,” where apparent progress slows, but gains are more robust because they are based in a deeper understanding. DBS for psychiatric indications may now be climbing that slope. The first round of trials were limited by the heterogeneity of depressive illness, poorly understood mechanism(s) of action, and limitations of a common “front end” trial design. Now, however, improved implant and laboratory-based technologies allow direct measurement of DBS' physiologic effects. These new technologies are emerging in the context of a major change in psychiatric nosology, a change that is expected to yield more reliable physiologic markers of mental illness. These two developments, taken together, have great potential to move psychiatric neuromodulation forward. We may soon see trials of closed-loop DBS systems, where the implant self-adjusts stimulation to bring a target biomarker to a pre-specified level associated with healthy function.

## Development and initial trials of DBS for depression

**Figure 2:**
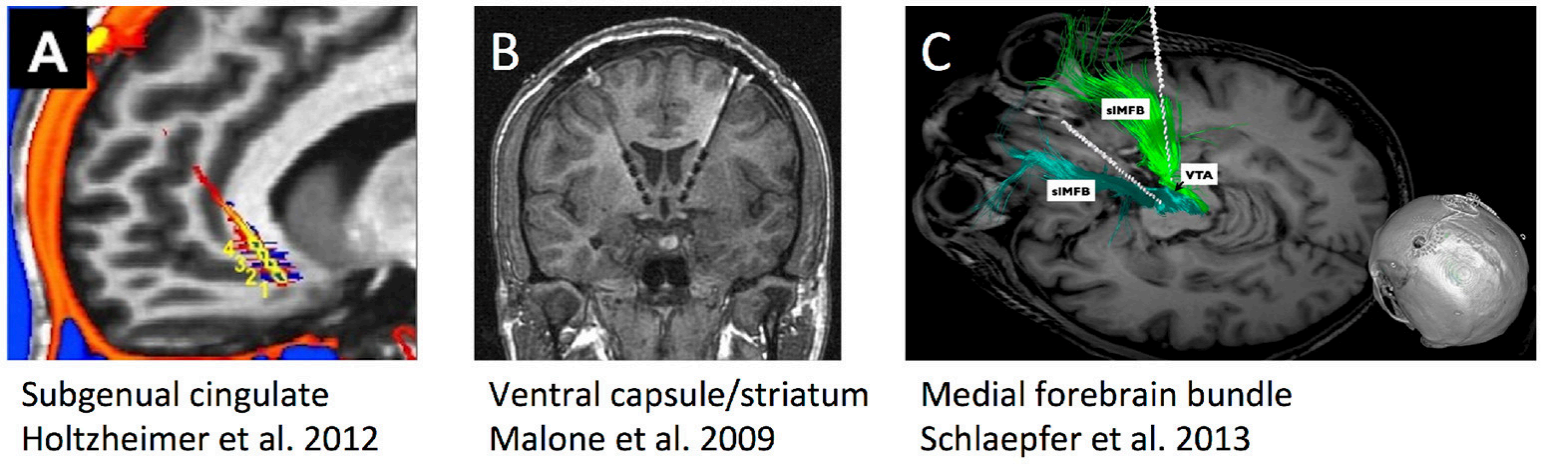
Schematic illustrations, re-used with permission, of the three DBS targets that have undergone human trials for treatment-resistant depression. **(A)** Subgenual cingulate gyrus (Cg25). **(B)** Ventral internal capsule/ventral striatum (VC/VS), also sometimes called anterior limb of internal capsule (ALIC). **(C)** Supero-lateral medial forebrain bundle (MFB).

Three DBS targets for TRD have each been tested in more than one center (Figure 2), each highlighting a different approach to target identification. The ventral capsule/ventral striatum (VC/VS) was a serendipitous finding during OCD DBS trials, where mood improvement preceded change in core OCD symptoms. Brodmann area 25 in the subgenual cingulate gyrus (Cg25) was identified in imaging studies of sad/negative mood and depressive illness. Superolateral medial forebrain bundle (MFB), by contrast, was based on theories of depression as a reward/motivation imbalance. Inclusion criteria have been similar across studies (Box 1), permitting qualitative comparisons.

Box 1 **Summary of inclusion/exclusion criteria across MDD DBS trials. MADRS, Montgomery-Asberg Depression Rating Scale. HAMD, Hamilton Depression Rating Scale. ECT, Electro-convulsive therapy.**

**Inclusion**

- Severe depressive episode: MADRS ≥ 26/HAMD-17 ≥ 18/HAMD-24 ≥ 21
- Non-response to at least 4 gold-standard treatments, including multiple medication classes and usually requiring ECT and/or adequate psychotherapy
- Medical stability for neurosurgical procedures
- Cognitive capacity sufficient for informed consent

**Exclusion**

- Psychotic disorders
- Recent history of suicidality or suicide attempt
- Active substance use, eating, or trauma-related disorders
- Severe personality disorders in many (not all) studies
- Bipolar disorder for VC/VS target (with rare exceptions)

### Serendipity

DBS developed when functional neurosurgeons noted that high-frequency electrical stimulation mimicked the effect of lesioning the same brain area (Gardner, 2013; Miocinovic et al., 2013). Since internal capsule lesions were a known therapy for OCD and MDD (Park et al., 2006), DBS was attempted in the anterior capsule, with Nuttin et al. (1999) publishing the first successful case series in OCD. As experience grew, investigators noticed that OCD patient's mood improved before their obsessive symptoms (Goodman et al., 2010; Greenberg et al., 2010). Imaging studies suggested that DBS of VC/VS and the neighboring nucleus accumbens (NAcc) modulates a broad fronto-limbic network (Figee et al., 2013; Haber and Heilbronner, 2013; Dougherty et al., 2016), including Cg25 (see below). Multiple groups thus piloted open-label stimulation of VC/VS and neighboring structures for MDD (Schlaepfer et al., 2007; Malone et al., 2009; Bewernick et al., 2010). In the largest published study, half of patients (8/15) responded with a 50% or greater drop in the Montgomery-Asberg Depression Rating Scale (MADRS) at the 3 month follow-up (Malone et al., 2009). The response rate was also 50% in a parallel *n* = 10 European trial, although this was only reported at 1 year (Bewernick et al., 2010). These studies also revealed that VC/VS DBS carries a risk of hypomania, even in patients without prior spontaneous or drug-induced bipolar features. The incidence is up to 50% over the course of DBS treatment, and is not readily predicted by patient or stimulation characteristics (Widge et al., 2015).

Those encouraging results led to the pivotal RECLAIM trial, sponsored by Medtronic. RECLAIM's primary outcome was MADRS change at the end of a 16-week blinded period. Before the blind, all patients received bilateral VC/VS DBS implants. All underwent initial “optimization,” searching stimulation settings to find those that caused acute mood effects. Half the patients then had their devices de-activated for 4 months; this blind was verified to be effective (Dougherty et al., 2015). An interim analysis at *n* = 30 showed no separation between active and sham stimulation at that 4-month timepoint, either on the continuous MADRS outcome (8 points active, 9.1 points sham) or response rate (3/15 active, 2/14 sham among completers). RECLAIM was thus ended early for futility. 24-month follow-up showed that patients who kept their DBS had a 23% (7/24) response rate, with the majority (6/7) of responders achieving remission.

A European study, conducted roughly in parallel, had a more encouraging result (Bergfeld et al., 2016). 25 patients received VC/VS DBS for TRD, with a 40% (10/25) response rate after a year of open-label therapy. Patients had a mean current-episode duration of 7 years, implying that spontaneous remission was unlikely during this open-label year. From the open-label phase, 16 patients (9 responders and 7 non-responders) entered a blinded cross-over where the DBS was de-activated in half the cohort at any given time. DBS discontinuation worsened depression in responders, but not in non-responders, and the difference met the pre-specified superiority threshold. This study was the first RCT of any invasive psychiatric therapy to reach its prospective success criterion.

### Imaging

The notion of DBS as mimicking a “reversible electrical lesion” spurred a question: rather than using historical lesion sites, could a DBS target be found through neuroimaging? Mayberg and colleagues pursued this through positron emission tomography (PET) and functional magnetic resonance imaging (fMRI). They found that the cingulate gyrus in Brodmann area 25 (Cg25) was hyperactive during sadness, in both depressed patients and volunteers undergoing mood induction (Mayberg, 2009). Open-label DBS of Cg25 in patients with TRD had a 66% response rate at 6 months in the pilot study and 55% at up to 6-year follow-up (Mayberg et al., 2005; Kennedy et al., 2011). Similar rates were seen in mixed cohorts of unipolar MDD and bipolar illness, supporting the claim of specificity for negative mood (Holtzheimer et al., 2012). St. Jude Medical Neuromodulation (now part of Abbott Laboratories) initiated the multicenter BROADEN study of Cg25 DBS based on these results. BROADEN also did not meet its primary endpoint (Holtzheimer et al., 2017). Newer results from the Mayberg group suggest that the effective target is not Cg25 itself, but a confluence of white matter tracts that sometimes occurs at this same point (Riva-Posse et al., 2017). The effects of stimulating this “affective hub” have not yet been replicated outside the original investigators, and we do not yet know how much of the response can be attributed to the intense and attentive research protocol at that site. That said, the tracts identified by Riva-Posse et al. pass through VC/VS and through other sites that have been effective in lesion surgeries for MDD, suggesting that Cg25 does access a “depression circuit” (Mayberg, 2009; Haber and Heilbronner, 2013; Makris et al., 2015).

### Theory

Anhedonia is a core feature of depression, and is often inadequately addressed by cognitive therapy or medications. Profound anhedonia may define a sub-class of treatment-resistant MDD with a particularly strong negative expectancy effect. Schlaepfer and colleagues theorized that by modulating reward pathways, they could treat highly anhedonic MDD patients. In support of this, they noted that VC/VS DBS often stimulates the nucleus accumbens (NAcc), a key node in that circuitry (Schlaepfer et al., 2007; Bewernick et al., 2010). They reasoned that, similar to the recent Mayberg group results, greater efficacy might come from modulating multiple reward sites at once through a white matter hub. The MFB is one such hub. The Schlaepfer group developed novel imaging techniques to locate MFB in individual patients, as white matter tracts have highly variable locations between individuals (Schlaepfer et al., 2013; Makris et al., 2015). In an open-label 7-patient series of bilateral MFB DBS, 6/7 experienced a 50% MADRS improvement by the last reported timepoint, which ranged from 12 to 33 weeks (Schlaepfer et al., 2013). Unlike Cg25 and VC/VS, MFB stimulation had very rapid effects on patient's self-ratings, with 4/7 reporting 50% improvement with less than a week of DBS. Although the target was proposed specifically for anhedonia, the effects were not specific to that symptom. Multiple aspects of the MADRS improved, as did anxiety rating scales.

The main limitation, unique to this target, was visual. MFB lies near oculomotor tracts, and stimulation at usual DBS currents impaired visual function in all 7 subjects. This may not be a limitation in practice, since lower currents still produced dramatic clinical results. Both VC/VS and Cg25 were similarly impressive in their open-label trials, and MFB may only have reached the peak of Figure 1. For example, a US group recently reported a planned single-blind replication of the German results. After 1 month with DBS electrodes and no stimulation, 2/4 patients already met the 50% MADRS response criterion (Fenoy et al., 2016). Activating DBS did produce a dramatic effect in 1 of the 2 sham non-responders, and both sham responders had further MADRS decreases at 1 week of stimulation. Similar to the prior targets, much work remains to differentiate MFB DBS' actual effects from placebo effects.

The mixed RCT results brought DBS for TRD firmly into the “valley of disillusionment,” raising questions about the value of further study. We remain optimistic about DBS' prospects in depression—but only if the community exploits the lessons learned from these “failed” trials. In the next section, we review those lessons and their potential implications.

## Scientific lessons from clinical-endpoint “failures”

These RCTs of DBS for TRD did not meet their endpoints, but they are not scientific failures. They revealed unique challenges of DBS work, including a remarkable placebo response, trial design concerns, and barriers to long-term follow-up. They provided evidence that there is a signal beyond placebo, but that this signal requires further refinements to capture. Most of all, the first round of trials gave us a sufficient patient base to study how DBS affects the brain, a critical design input for closed-loop systems.

### DBS involves multiple strong placebos

First, many DBS studies are small open-label trials where a center is attempting an implant or stimulation technique for the first time. DBS thus suffers from the “non-specific therapeutic effects” problem, where patients recover in part because of intense clinical attention. This is worse than usual with DBS because placebo responses increase with the cost and invasiveness of an intervention (Espay et al., 2015). For instance, the RECLAIM sham response of 14% is quite large for TRD. Second, because of the frequent follow-up and complication risks, many centers encourage patients to relocate close by before implant. The move may “reset” maladaptive behavior patterns and/or serve as therapeutic behavioral activation. Third, an electrically inactive (sham) DBS lead is still a biological intervention. DBS has a well-established “micro-lesion effect,” where a foreign body response causes hypo-function of the implanted nucleus for weeks to months (Miocinovic et al., 2013). Something similar likely occurs in psychiatric patients, and small lesions are known to have beneficial mood effects (Park et al., 2006).

That said, there is reason to believe DBS has effects beyond placebo. Three key pieces of evidence support this: strong psychological side effects, relapses from blinded discontinuation, and physiologic changes. First, DBS at psychiatric targets can have dramatic side effects that are hard to label as placebo/nocebo. VC/VS stimulation causes hypomania (Widge et al., 2015), while Cg25 causes a similar emotional “rough patch” (Crowell et al., 2015). Hypomania in particular depends on the stimulation intensity, a dose dependence we should not see with a nocebo effect. Second, blinded discontinuations cause relapses. The Bergfeld study used this to demonstrate its endpoint, but patients also have inadvertent, blinded discontinuation when a device component fails. Across studies, this led to depressive relapse even though each patient was completely unaware that his/her device was non-functional (Holtzheimer et al., 2012; Dougherty et al., 2015). The same data argue that DBS' effects are not explained by spontaneous remission of the depressive episode. An endogenous remission/relapse would not track with active/inactive DBS. Third, non-invasive imaging and neurophysiology studies show brain changes specifically in response to active DBS, both at the target and at connected cortical structures (Mayberg et al., 2005; Broadway et al., 2012; Figee et al., 2013; Bahramisharif et al., 2016; Dougherty et al., 2016; Widge et al., 2016b). Those small-sample studies are not entirely consistent in their findings, but they provide evidence for an effect beyond the micro-lesion or a patient expectancy. The limited sampling resolution of these imaging studies (capturing patients at a single point in time post-treatment) also highlights another reason to move toward physiology-based, closed-loop DBS. With longitudinal sampling over time, the relation between brain changes and physiologic response might become more evident.

### Detecting clinical signal requires extended treatment

The two DBS for TRD trials that did not meet their primary endpoint had a similar “front end” design: implant, sham vs. active stimulation, then open-label treatment, with the key outcome being the sham/active difference after 4–6 months of stimulation. The only DBS trial that met its prespecified endpoint for TRD started all patients on open-label treatment, then performed a blinded discontinuation at roughly 1 year (Bergfeld et al., 2016). This is similar to vagus nerve stimulation (VNS), where the value of active stimulation is only visible after extended treatment (Aaronson et al., 2017). MFB DBS might be testable with the front-end design, but the strong sham effect in the Fenoy et al. (2016) study argues against that.

Given the difficulties with the front-end approach, we believe future DBS studies should emphasize the “back end” blinded discontinuation approach, in both responders and non-responders. By potentially amplifying the sham/active difference or enabling a within-subject comparison, back end discontinuation also addresses a statistical power concern. All published DBS for TRD trials were powered to detect very large active/sham differences. The back end design amplifies those differences by providing a longer window to optimize treatment settings. This design may also control better for inter-site variability in large RCTs. If one site is using a variant surgical or programming approach that impairs efficacy, this will be much more apparent with a long open-label lead in, and may be correctable in the analysis. That inter-site variation would also become more evidence in closed-loop studies, where the outlier site would presumably have difficulty changing the target biomarker.

### Sites must be prepared for long-term care

That long treatment duration, combined with the severity of patient's illness, creates unique challenges for DBS in MDD or other psychiatric disorders. First, each implant is a major commitment to the patient. DBS still has idiosyncratic risks. The design of most implants (with power wires passing through the neck to a chest or abdominal battery) creates many opportunities for device failures in young, physically active patients. Friends, family, and even other psychiatrists tend to attribute any change in mental status to the device (Klein et al., 2016; Goering et al., 2017). Each patient requires substantial psychiatrist, surgeon, and support staff time. Study funds rarely will completely cover these costs. Further, there may be years between the end of a successful trial and formal regulatory/payment approval. Individual patients from unsuccessful trials often want to keep their devices, and the costs of their follow-up are borne by the investigators. These burdens led one of the RECLAIM sites to transfer or explant all of its patients at the study conclusion (Dougherty et al., 2015). Teams need to understand the time and financial commitment well before a study opens, and have a long-term plan in place. This will be doubly true if continued use of the system requires technical expertise in engineering and/or electrophysiology. In fact, NIH now requires a written long-term care plan as part of applications for advanced neurotechnology studies.

### Symptom heterogeneity may cloud trial results

**Figure 3:**
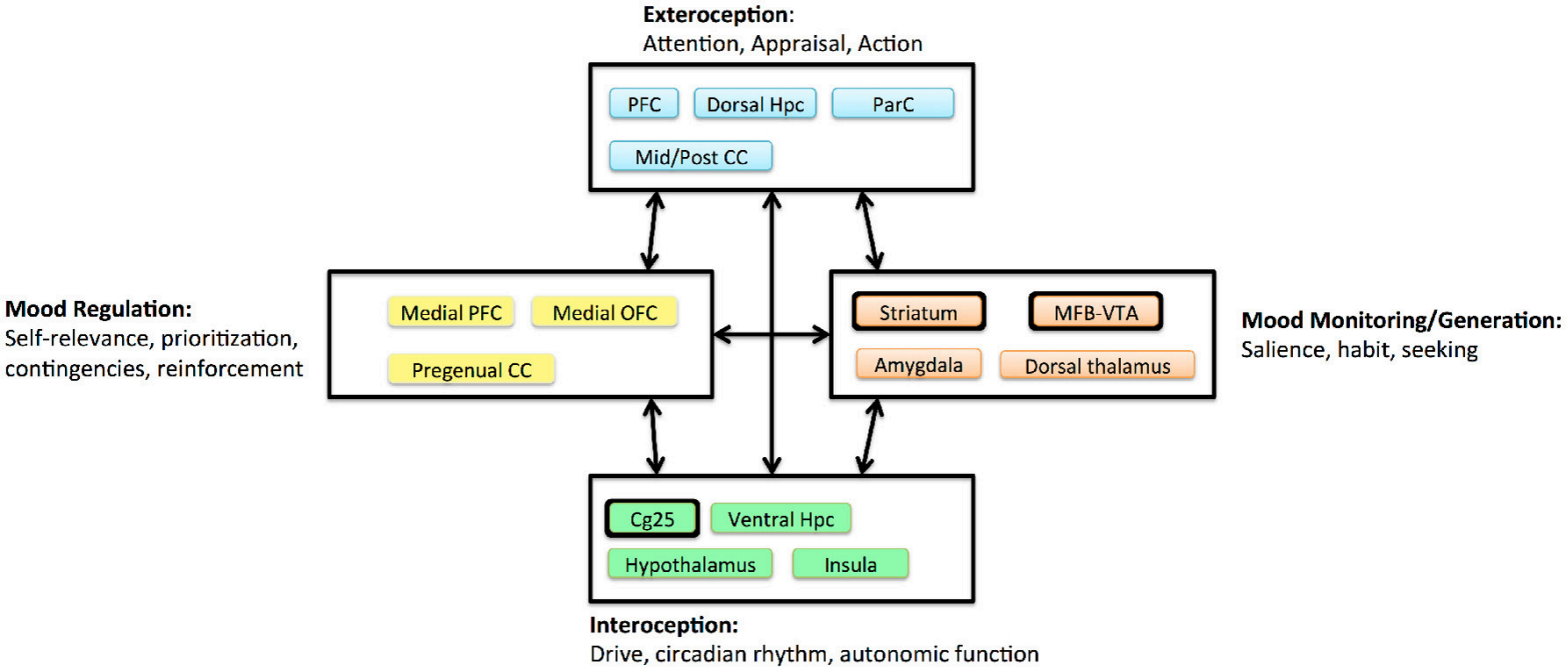
Circuit model of MDD proposed by Mayberg (2009), adapted and simplified to emphasize DBS effects. Boxes denote clusters of structures with evidence for tight anatomic inter-connectivity and relevance to a sub-domain of impairment within MDD. Arrows denote long-range interactions believed to exist between these subnetworks. Major DBS targets are highlighted with thickened outlines. The three extant targets all project to a broad prefrontal network, and all are structures believed to generate more “primitive” internal experiences. CC, cingulate cortex; Hpc, hippocampus; MFB, medial forebrain bundle; OFC, orbitofrontal cortex; ParC, parietal cortex; PFC, prefrontal cortex; VTA, ventral tegmental area.

One key lesson from the first-wave trials is that we do not know exactly which disease DBS treats. MDD is prevalent because it is a heterogeneous disorder, with many symptom combinations that qualify a patient for the diagnosis. TRD in particular may include undiagnosed medical, personality, or comorbid psychiatric illness. The three extant DBS targets access the same fronto-limbic circuit (Figure 3), but may modulate it in very different ways. Even acute stimulation at each target has different effects: a sense of lightness at Cg25, anxiety relief at VC/VS, and a hedonic seeking/wanting at MFB (Mayberg, 2009; Greenberg et al., 2010; Schlaepfer et al., 2013; Crowell et al., 2015). Multiple investigators have suggested that each target is likely most effective for a specific sub-group of symptoms, such as a predominance of affective dysregulation vs. affective flattening (Crowell et al., 2015; Widge et al., 2016a).

### Commercial viability requires greater reliability

The concept of fractionating patients by phenotype or biological impairment is part of a broader theme: to survive in the coming era of value-oriented medicine, DBS for TRD or any other psychiatric indication must be more reliable and predictable in its effects. A DBS implant costs about $64,000 for the surgery and devices, and about another $3,600 annually for device maintenance (Stroupe et al., 2014). TRD patients rate their quality of life about 12% worse than treatment-responsive MDD patients (Mrazek et al., 2014). As a back-of-the-envelope calculation, assuming the 40% response rate of the Bergfeld et al. trial, 15 years of DBS treatment, no relapses, and no effect on mortality, DBS has a cost per quality-adjusted life year (QALY) of about $165,000. This is well above the estimated $23,000/QALY of DBS for Parkinson disease (Pietzsch et al., 2016), the commonly-used standard of $50,000/QALY, and even some more generous thresholds that have been suggested (Neumann et al., 2014). Society might support $100,000/QALY if decision-makers consider the indirect burden of TRD on caregivers and employers. To reach that threshold, DBS must achieve a response rate closer to 70%, ensure that most responders are also remitters, and/or lower its cost. Those same steps would address patient's primary frustration with psychiatric DBS: its uncertainty. In a series of patient interviews, both responders and non-responders were specifically unhappy with the trial-and-error programming process (Klein et al., 2016; Goering et al., 2017). This again suggests closed-loop technologies as a way forward. Titrating stimulation against a physiologic marker should be clinically more effective, but will also build patient's confidence in their treatment.

## Toward closed-loop DBS: emerging insights and platform technologies

To “climb the slope of enlightenment” and develop those closed-loop technologies, we must first identify a physiologic target. This is the major technical barrier to closing the loop: we do not yet understand how successful DBS changes a patient's brain. Three lines of research are developing that understanding: diagnostics and patient phenotyping, post-implant studies of DBS neurophysiology, and efforts toward novel stimulation paradigms. All three are leveraging new DBS system designs that give investigators a direct view into the patient's brain activity. Taken together, they suggest that closed-loop trials are possible in the near-term future.

### Phenotyping and diagnostics

DBS acts much more focally than other common psychiatric treatments, e.g., medications or convulsive therapy. DBS might thus not be effective for “TRD” generally, but only for patients whose MDD arises from deficits in the stimulated circuit. There is already an anecdotal sense that specific clinical phenotypes are more likely to respond to DBS at specific targets, e.g., profound anhedonia to MFB or emotional dysregulation to VC/VS (Schlaepfer et al., 2013; Crowell et al., 2015; Widge et al., 2016a). The challenge is that subjective, anecdotal impressions do not create closed-loop biomarkers. Instead, these intuitions must be turned into quantitative metrics and regressed against physiology. This would effectively be a new science of psychiatric diagnosis, based not on symptom clusters, but on directly measuring circuit impairments (Insel, 2010; Gordon, 2016).

Large initiatives such as iSPOT-D (https://med.stanford.edu/williamslab/research/complete/ispotd.html), EMBARC (http://embarc.utsouthwestern.edu/), and TRANSFORM (https://transformdbs.partners.org/) are working to identify such circuit phenotypes, and have shown preliminary success (Etkin et al., 2015; Grisanzio et al., 2017; Widge et al., 2017). The potential value of that work is evident when considering the history of DBS for movement disorders. DBS' original proposed indication was chronic pain, but pain rating scales (like depression scales) are extremely subjective, making it harder to show efficacy (Gardner, 2013). The approval for Parkinson disease depended in part on validating an objective tool, the Unified Parkinsons Disease Rating Scale (UPDRS), which improved rater reliability (Gardner, 2013). Improved reliability, in turn, enabled the physiologic studies linking beta-band neural oscillations to Parkinsonian symptoms (see below). Better phenotyping systems could do the same for psychiatry and for MDD in particular.

The next step would be ensuring that we engage the target circuit in every patient. Most psychiatric DBS modulates circuits through white matter tracts. The location of target tracts varies substantially between patients, and multiple fiber bundles are often interwoven (Makris et al., 2015). Recent work, much of it driven by the Mayberg group, has shown that computational models can help clinicians shape the DBS electrical field to target specific sub-bundles (Noecker et al., 2017; Riva-Posse et al., 2017). Early open-label and retrospective reports specifically suggest that this may increase DBS' efficacy in TRD (Riva-Posse et al., 2014, 2017). An advanced DBS device to support more precise field-shaping is already available in Europe, and there is clinical evidence that a shaped field reduces side effects (McIntyre et al., 2015). This model-based field shaping could be further augmented by physiologic monitoring. We could ensure not only that the correct circuit is stimulated, but that the stimulation changes brain activity in a desired way.

### Connecting anatomy to physiologic effects

Electrical brain stimulation, by definition, alters neuron's signaling. Studies in Parkinson disease suggest that DBS affects rhythmic neural firing patterns that may coordinate distributed brain networks. Chief among these is a finding that beta (15–30 Hz) oscillations are linked to akinesia and rigidity (de Hemptinne et al., 2015; Quinn et al., 2015; Swann et al., 2017). This was both a large step toward closed-loop DBS and a demonstration of the value of sensing/recording closed-loop implants. Without long-term intracranial human recordings, the beta marker would have been difficult to link to long-term symptoms.

Electroencephalography (EEG) can extract similar signals from cortical regions, and has frequently been used to study depression (Wade and Iosifescu, 2016). A few studies have used EEG to measure DBS' physiologic effects and/or correlate those effects to clinical symptoms (Broadway et al., 2012; Bahramisharif et al., 2016; Widge et al., 2016b). Unfortunately, none has yielded a reliable biomarker, and in general, EEG-based biomarkers of MDD neurostimulation response have not replicated well (Widge et al., 2013, 2016b; McLoughlin et al., 2014; Wade and Iosifescu, 2016). This likely arises from MDD's heterogeneity, as discussed above. Each of those EEG studies may have inadvertently measured a different disorder. Identifying more reliable biomarkers, e.g., via the circuit-oriented approach also reviewed above, would be a pre-requisite for closed-loop psychiatric DBS. The TRANSFORM project has validated a preliminary version of this circuit approach using invasive recordings in non-psychiatric volunteers (Widge et al., 2017).

### Platforms for longitudinal physiologic monitoring

As also discussed above, DBS in MDD requires extended treatment to demonstrate clear effects. In Parkinson disease, DBS' effects appear and disappear within seconds. Mood and anxiety effects from DBS take weeks to months (Malone et al., 2009; Mayberg, 2009; Dougherty et al., 2015; Bergfeld et al., 2016; Riva-Posse et al., 2017), and even the allegedly rapid-acting MFB target shows a long-term improvement phase (Schlaepfer et al., 2013; Fenoy et al., 2016). Even if our proposed circuit-oriented phenotyping improves signal-to-noise ratios, it may be difficult to identify biomarkers from laboratory-based recordings. If individual patients progress toward response at different rates, studies that capture their brains at single timepoints (or with months between recordings) are likely to miss critical changes. A better strategy would be to record repeatedly, and at high temporal density, over a year or more of continued DBS treatment.

To collect human neurophysiology at those timescales, manufacturers have developed DBS systems with data recording and storage capabilities, reviewed in depth in Lo and Widge (2017). Medtronic's PC+S system can sense brain signals during continuous stimulation and can operate in free-moving humans for years. PC+S is validated for recording cortex and deep brain simultaneously (Swann et al., 2017), a configuration that might be helpful for monitoring psychiatric circuit function. Neuropace also makes a recording-capable stimulator, and their device is FDA-approved for seizure control (Bergey et al., 2015), while the Medtronic PC+S is only available under an investigational protocol. The Neuropace device is specifically designed for closed-loop operation, and is arguably the first example of a commercially successful bi-directional BCI. On the other hand, PC+S has been successfully used for closed-loop tremor control in laboratory settings (Little et al., 2013; Herron et al., 2016).

Both Neuropace and the Medtronic PC+S are currently being used to collect longitudinal electrophysiologic recordings in patients being treated with DBS for psychiatric indications (clinicaltrials.gov NCT01329198, NCT01984710, NCT02056873, NCT03184454, among others). Those pilot studies are still based around traditional diagnoses rather than circuit/dimensional constructs. They should nevertheless yield important information about how the brain responds to DBS-like stimulation. The slow timescale of clinical response suggests that neuroplasticity plays a role in DBS for TRD (Herrington et al., 2015). Daily-to-weekly recordings might make those plasticity processes visible for the first time. The enthusiasm for the PC+S and Neuropace devices has also led to projects developing novel implants optimized for high-channel-count recording and stimulation (Wheeler et al., 2015; Bjune et al., 2016; Moin et al., 2016; Zhou et al., 2017). If those technologies progress to clinical viability, we will have unprecedented views of the human mind in health and dysfunction.

### Improved efficacy from next-generation technologies

We can also anticipate more effective stimulation approaches. For instance, existing DBS devices can stimulate more than one site simultaneously, which may be a better approach for circuit intervention. Dual-site cortical stimulation had preliminary success in MDD (Nahas et al., 2010). It may also be possible to achieve DBS' effects with non-invasive technologies. Transcranial focused ultrasound (TFUS) can modulate deep structures (Fini and Tyler, 2017), albeit with slow onset/offset. TFUS also can be used in place of open neurosurgery to create brain lesions (Ghanouni et al., 2015). As noted above, lesions can be as efficacious as DBS and may offer greater patient convenience in some situations. TFUS-based lesioning may develop as a complement to DBS. A new technique, temporal interference stimulation, might also offer depth-specific non-invasive stimulation with better time resolution (Grossman et al., 2017). These are prototype technologies, but they make a case that DBS may not always be limited to implantable devices or continuous high-frequency energy. With non-invasive technologies, it will be even more important to have a means of monitoring the brain's response to energy delivery. Variations in human cranial anatomy can shunt energy away from its nominal target, and monitoring physiologic response would be the best way to confirm target engagement.

## Conclusions

Invasive neuromodulation, and DBS in particular, still has promise for treating psychiatric illness. Although the first randomized clinical trials did not produce their desired results, some patients experienced life-transforming benefits, and there is evidence for a signal beyond placebo. Those trials also identified numerous barriers to wider use of DBS in MDD/TRD. Chief among these is the subjective, trial-and-error nature of DBS programming, especially when combined with the weeks to months needed to detect clinical effects. In recent years, however, recognition of those barriers has spurred efforts to understand the physiologic basis of DBS response. Those have dovetailed with a broader effort to re-orient psychiatric neuroscience to a systems/circuits perspective. DBS is particularly well-suited to that mode of thought because stimulation acts on an anatomically limited circuit. Preliminary biomarkers of circuit dysfunction have been published. At the same time, advanced DBS hardware is making it possible for investigators to target those biomarkers in a closed-loop fashion. Advanced hardware is also allowing multiple groups to study DBS' effects in a dense, longitudinal way. In short, all the pieces of a closed-loop therapy for TRD exist as prototypes. They still must travel a long road of validation/replication, integration, and testing in real-world patient populations. Establishing that evidence base is the slow but monotonic “slope of enlightenment” in Figure 1.

## Author contributions

All authors listed have made a substantial, direct and intellectual contribution to the work, and approved it for publication.

### Conflict of interest statement

AW and DD receive consulting income and device donations from Medtronic. DD and DM have also received research funding from Medtronic. DD further reports consulting income from Insys, speaking fees from Johnson and Johnson, and research support from Cyberonics and Roche. AW and DD are inventors on multiple patent applications related to deep brain stimulation.

## Footnotes

^1^Gartner, Inc. Hype Cycle Research Methodology. Available at: http://www.gartner.com/technology/research/methodologies/hype-cycle.jsp (Accessed September 27, 2016).
